# Comparative analysis of the color change in blue-green inclusions within neutrophils between two patients with different clinical outcomes

**DOI:** 10.11613/BM.2024.030801

**Published:** 2024-08-05

**Authors:** Junkun Chen, Ming Huang, Guo Li, Chi Zhang

**Affiliations:** Department of Laboratory Medicine, Tongji Hospital, Tongji Medical College, Huazhong University of Science and Technology, Wuhan, People’s Republic of China

**Keywords:** inclusion bodies, liver failure, bunyavirus infection, wasp sting, patient-relevant outcome

## Abstract

Blue-green neutrophilic inclusions (BGNI), also known as “death bodies,” are bright green structures observed in the cytoplasm of neutrophils or monocytes and are closely associated with acute liver failure, lactic acidosis, and other serious diseases. Some studies suggested a potential association with phagocytic lipofuscin released by damaged liver cells. The presence of BGNI typically indicated a poor prognosis. We presented two cases. Case 1 was diagnosed with novel bunyavirus infection and exhibited severe hepatic impairment and coagulation dysfunction along with the presence of BGNI in neutrophils. Despite receiving comprehensive symptomatic treatment, the patient’s condition rapidly deteriorated leading to eventual demise. Case 2 had severe liver injury caused by wasp stings, and BGNI was observed. Following active treatment measures, the patient eventually achieved recovery. Throughout the disease course of case 1, there was a progressive deepening in color and increase in quantity of BGNI. Conversely, case 2 demonstrated an opposite trend. Based on the comparison of clinical outcomes and variations in color and quantity of BGNI between these two patients, it was found that an increase in the number and deepening of BGNI color corresponded to worsening condition. Conversely, a decrease in quantity and lightening of color indicated improvement. Hence, these findings suggest a possible association between changes in BGNI characteristics and prognosis.

## Graphical abstract

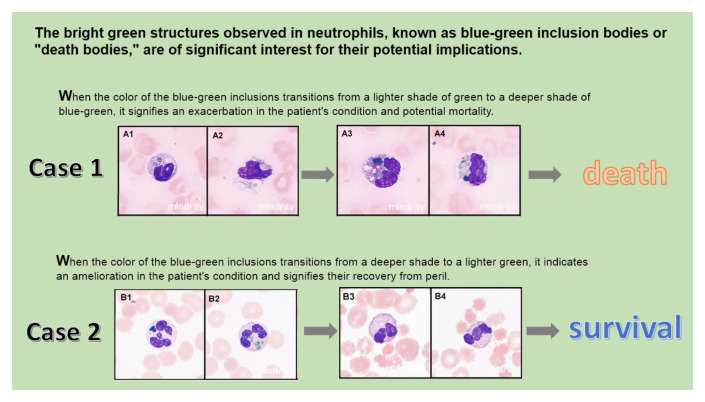

## Highlights

• Blue-green inclusion bodies are associated with severe conditions such as liver failure and poor prognosis

• The report describes two cases of the inclusions: one from bunyavirus infection and the other from wasp sting

• Comparing cases, we speculated on a correlation between the inclusions and patient-relevant outcomes

## Introduction

Blue-green neutrophilic inclusions (BGNI), bright green structures in the cytoplasm of neutrophils or monocytes, are also known as “death bodies”, which are closely related to acute liver failure, lactic acidosis and other critical diseases (*1*). Since the first discovery of this phenomenon in peripheral blood smears (PBS) of patients with acute liver failure, more and more laboratory technologists have found the presence of BGNI in peripheral blood neutrophils of various critically ill patients. The special inclusions has been widely reported in acute liver failure, yellow fever, bacteremia, sepsis, COVID-19 infection, severe fever with thrombocytopenia syndrome (SFTS), and fungal and plasmodium infection (*2*-*8*). Although Harris *et al*. speculated that BGNI was related to blood-derived biliverdin, this hypothesis has not been confirmed (*2*). Hodgson *et al.* demonstrated that these inclusions were mainly composed of lipid-rich substances by three different staining methods (*1*). They found that when stained with Giemsa’s solution, BGNI closely resembled lipofuscin in liver cells, a green lipid deposit found in liver, heart, kidney, retina, nerve cells, and adrenal cells. Its main component is a heterogeneous mixture composed of lysosomally digested lipid-containing residues, proteins, carbohydrates and trace elements (*9*). It is speculated that neutrophils may have formed this green inclusion by swallowing lipofuscin released by damaged liver cells. This further suggests that BGNI in PBS is closely related to severe hepatocyte injury. In addition, it is worth noting that in most of the reported cases, the patients were accompanied by symptoms of lactic acidosis, and the accumulation of lactic acid in the body would further aggravate the damage and destruction of tissue cells, resulting in the intensified release of lipofuscin and increasing the probability of BGNI (*10*). Therefore, the presence of such inclusions usually indicates a poor prognosis for the patient and may even be life-threatening in the short term.

## Case presentation

**Figure 1:**
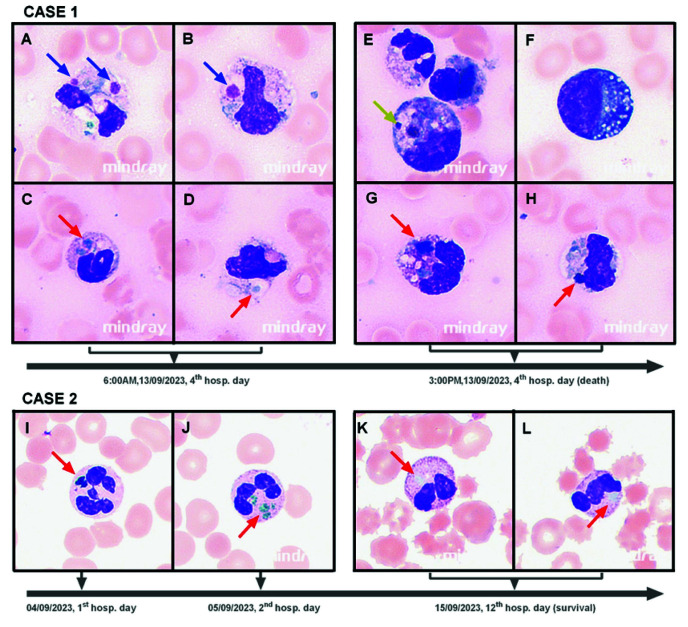
The dynamic changes of peripheral blood cell morphology in the two cases. Case 1: A and B signifies that at 6:00, the patient’s neutrophils not only phagocytosed platelets but also contained light-colored blue-green inclusions. C and D represent the observation of light-colored blue-green inclusions within the patient’s neutrophils at 6:00 on the day of death in Case 1. E indicates that Howell-Jolly bodies and deeply stained blue-green inclusions appear simultaneously in neutrophils at 15:00. F indicates the presence of plasmablast-like reactive lymphocytes observed in the patient’s blood sample at 15:00. G and H indicate a significant darkening of the blue-green inclusions within the neutrophils at 15:00 on the same day. Case 2: I signifies the presence of dark-colored blue-green inclusions within the patient’s neutrophils on the day of admission. J indicates that the color of these blue-green inclusions began to lighten on the 2nd day of admission. Finally, K and L represent the observation that, although blue-green inclusions were still present within the patient’s neutrophils on the 12th day of admission (which was also the discharge day), their color had become very pale.

We report two cases in which BGNI was found on PBS and their morphology was imaged using the Mindray MC-80 blood cell morphology analyzer, as shown in Figure 1: A1-A4,B1-B4.

**Figure 2:**
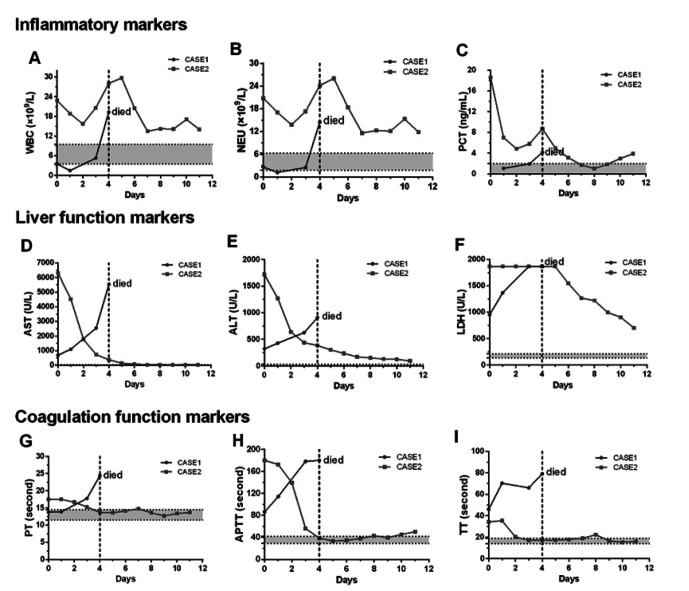
Dynamic chart of abnormal laboratory indicators in two cases. A-C, D-F and G-I represented inflammation, liver function and coagulation function indicators, respectively. The horizontal axis represented the length of hospital stay, and the vertical axis represented the level of various indicators. The darker line represented case 1 and the lighter line represented case 2. The dashed line on the X-axis indicated the time of appearance of the blue-green inclusions in Case 1, which was also the time of her death. The gray area on the Y-axis represented the normal reference interval of the indicators. WBC - white blood cell count. NEU - neutrophils. PCT - procalcitonin. AST - aspartate aminotransferase. ALT - alanine aminotransferase. LD - lactate dehydrogenase. PT - prothrombin time. APTT - activated partial thromboplastin time. TT - thrombin time.

Case 1 was a 58-year-old woman who had been suffering from chronic hepatitis B for more than 10 years and reported that she did not strictly follow the doctor’s advice and that the time of medication was irregular and intermittent. After being infected by novel bunyavirus, she was admitted to hospital with symptoms such as high fever, cough, phlegm, and diarrhea. Laboratory tests during admission revealed: the patient exhibited a relative decrease in peripheral blood cells, particularly a significant reduction in platelets. Liver enzyme activities were elevated, along with significantly increased serum ferritin concentrations and lactate dehydrogenase (LD) activity. Abnormal coagulation function was observed, as well as the presence of platelet phagocytosis (indicated by blue arrows in Figure 1: A-B) and plasma blast-like reactive lymphocytes (Figure 1: F). Various cytokine concentrations were also elevated, indicating signs of hemophagocytic syndrome and cytokine storm in the patient’s body. Analysis of peripheral blood lymphocyte subsets revealed decreased numbers of all lymphocyte subgroups except for an abnormal increase in plasma cell proportion, suggesting possible immune dysfunction due to viral invasion. Details are given in Table 1. The patient received a full range of symptomatic treatments including antiviral (Pofol tenofovir fumarate), anti-infective (biapenem + Teicoplanin), anti-inflammatory (dexamethasone 15 mg *qd*), continuous renal replacement therapy (CRRT), and stomach and liver care. However, the patient’s inflammatory indicators, liver function, and coagulation function were not controlled, but continued to deteriorate, as shown in Figure 2: A-I. When the patient was admitted, no BGNI was observed in the peripheral blood smear examination. However, on the 4th day of hospitalization, BGNI appeared in her peripheral blood (indicated by red arrows in Figure 1: C-D), accounting for 2% of neutrophils. On the second PBS sent later in the day, BGNI was still present and further deepened in color (indicated by red arrows in Figure 1: G-H) and increased in quantity (up to 5% of neutrophils). The patient’s condition deteriorated rapidly and she died within the same day.

**Table 1:** Laboratory examination indicators of two patients on admission

**Parameter (unit)**	**Result**		
	**Case1**	**Case2**	**Reference interval**
**Complete blood count**			
Leukocyte count (x10^9^/L)	3.59	**22.86**	3.5-9.5
Neutrophil count (x10^9^/L)	2.67	**20.82**	1.8-6.3
Lymphocyte count (x10^9^/L)	**0.57**	1.16	1.1-3.2
Percentage of monocytes (%)	9.2	3.6	1.0-10.0
Mean corpuscular volume (fL)	91.1	**79.1**	82-100
Mean corpuscular hemoglobin (pg)	31.2	29.2	27-34
Platelet count (x10^9^/L)	**18.0**	**72.0**	125-350
**Routine chemical testing**			
Aspartate aminotransferase (U/L)	**683**	**1717**	0-33
Alanine aminotransferase (U/L)	**322**	**6301**	0-32
Total bilirubin (umol/L)	14.3	**134.8**	≤ 21
γ- glutamyl transpeptidase (U/L)	97	**50**	6-42
Lactic dehydrogenase (U/L)	**952**	**> 1867**	135-214
Lactic acid (mmol/L)	**2.31**	1.95	0.5-2.20
Potassium (mmol/L)	4.56	**5.37**	3.5-5.1
Sodium (mmol/L)	134.1	127.7	135-145
Chloride (mmol/L)	104.2	95.3	99-110
Calcium (mmol/L)	**2.06**	2.11	2.15-2.5
High-sensitivity cardiac troponin I (pg/mL)	**77.5**	**426.9**	<15.6
Ferritin (µg/L)	**> 50,000.0**	NA	15-150
**Routine coagulation testing**			
Activated partial thromboplastin time (s)	**86.2**	**> 180**	29-42
Thrombin time (s)	**46.0**	**34.3**	14-19
D-D dimer (µg/mL FEU)	**3.80**	**2.07**	< 0.5
**Peripheral blood lymphocyte subsets**			
Total T lymphocytes (/µL)	**122**	NA	955-2860
Total B lymphocytes (/µL)	**79**	NA	90-560
Helper/inducible T lymphocytes (/µL)	75	NA	550-1440
Suppressor/cytotoxic T lymphocytes (/µL)	**38**	NA	320-1250
Natural killer cell (/µL)	**27**	NA	150-1100
Plasmocyte (/µL)	**31.9%**	NA	0.20-3.90%
**Cytokine detection**			
Interleukin-1β (pg/mL)	< 5.0	NA	< 5
Interleukin-2 receptor (U/mL)	**3594**	NA	223-710
Interleukin-6 (pg/mL)	**324.70**	NA	< 7
Interleukin-8 (pg/mL)	**93.0**	NA	< 62
Interleukin-10 (pg/mL)	**771.0**	NA	< 9.1
Tumor necrosis factor α (pg/mL)	**60.7**	NA	< 8.1
Abnormal data are highlighted in bold. NA - not available.			

Case 2, a 49-year-old woman, suffered severe wasp swarm stings on September 3, 2022, mainly on the head and upper arms, with a cumulative injury area of 185 cm^2^. Her wound was red and swollen with severe pain, followed by yellow skin and sclera, soy-colored urine, chest tightness, chest pain and nausea, so she was hospitalized. The results of the initial laboratory examination on admission revealed: the hemogram was elevated, liver enzymes, LD, and high-sensitivity cardiac troponin I were markedly elevated, and coagulation was abnormal; details of each indicator are provided in Table 1. In the PBS morphological examination on the day of admission, dark blue-green, round and large BGNI in neutrophils were found (indicated by red arrows in Figure 1: I-J), accounting for 3% of neutrophils. After notifying her attending doctor by phone, the patient immediately received symptomatic treatment such as bedside hemodialysis, ventilator assisted ventilation, anti-infection, blood transfusion and fluid replenishment, liver and stomach protection, and her condition gradually improved. After 12 days, most of the test indicators of the patient gradually returned to normal, as shown in Figure 2: A-I. Although BGNI are still visible in PBS, they have become significantly lighter in color (they are still round and appear light green, indicated by red arrows in Figure 1: K-L) and their proportion has decreased (1% of neutrophils). As her condition stabilized, she was assessed by doctors and discharged the following day.

Blood samples were collected from both patients on admission, and relevant examinations were carried out in time. The concentrations of interleukin-1β, interleukin-2 receptor, interleukin-8, interleukin-10 and tumor necrosis factor α were detected according to an automatic procedure of a solid-phase two-site chemiluminescent immunometric assay *via* IMMULITE 1000 Analyzer (Siemens Healthcare Diagnostics Products Limited, Llanberis, UK). The concentration of interleukin-6 was measured by electrochemiluminescence method (Roche Diagnostics, Mannheim, Germany). The number of lymphocyte subsets was measured by flow cytometry *via* BD FACSLyric (Becton, Dickinson and Company BD Biosciences, San Jose, USA). Informed consent was obtained from the patients or family member according to the Declaration of Helsinki and the study was approved by the ethics committee of Tongji Hospital (Wuhan, China).

## Discussion

Both cases showed BGNI in peripheral blood smears. However, there were significant differences between them. In the first case of the patient, the color of BGNI was gradually deepened and the number increased in the PBS. In contrast, in the second case, BGNI gradually became lighter in color and decreased in number over the course of the disease. The two patients ended up with very different outcomes.

**Figure 3:**
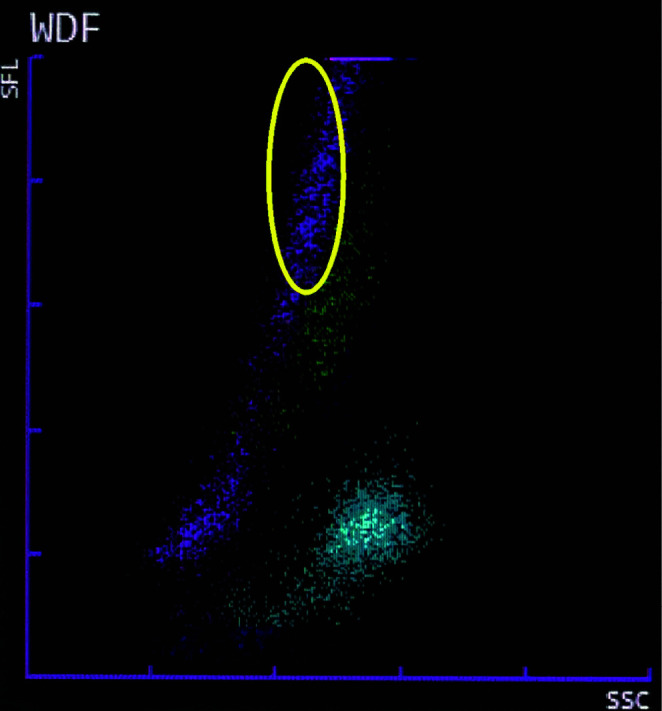
White blood cells classification scatterplot measured by flow cytometry *via* SYSMEX XN-20 for the case 1. Yellow oval frame represents the cluster of reactive lymphoid cells.

Novel bunyavirus is mainly transmitted by tick bites, and it first invades lymphocytes (especially B cells), causing lymph node enlargement and necrotizing lymphadenitis in the bite area (*11*). Patients infected with the virus experience decreased platelets and white blood cells, as well as fever, fatigue, muscle pain, gastrointestinal tract and other symptoms (*12*). Currently, there are no specific drugs, and patients are mainly given symptomatic and supportive treatment. After infection with the virus in immunocompromised patients, novel bunyavirus can highly replicate in the body, causing viremia, activating immune cells, causing cytokine storms, severe inflammatory reactions, hemophagocytic syndrome, *etc*. Eventually, the patient may die from multiple organ failure (*13*). After the patient in Case 1 was infected with novel bunyavirus, her aspartate aminotransferase (AST) and alanine aminotransferase (ALT) activities increased rapidly over a short period of time (Figure 2: D-E). The possible reason was that the patient had a history of chronic hepatitis B and had not received formal treatment, so her liver function and immune function were poor. Her disease quickly progressed to liver failure and BGNI appeared in her peripheral blood. The results of lymphocyte subsets suggested that the patient’s body was in a state of high exhaustion of T cells and immune response imbalance. At the same time, there was a cluster of cells (Figure 3, yellow oval box area) in the high-fluorescence region of the white blood cell scatter diagram, which usually represented the reactive lymphocytes. Microscopic examination also confirmed the presence of a high proportion of plasma blast-like reactive lymphocytes in the peripheral blood (Figure 1: F), which was consistent with the results of flow cytometry. Literature had shown that SFTS patients with higher plasma cell number were more likely to die in a short period, so high plasma cell number predicted poor prognosis of patients (*14*). In addition to the above, increased cytokine and lactate concentrations also indicated the presence of cytokine storm and lactate poisoning in patients. In addition, her peripheral blood also showed the phenomenon of neutrophils phagocytosis of platelets, and her ferritin was also increased, suggesting that the patient had hemophagocytic syndrome. At the same time, we also found Howell-Jolly body-like inclusions in the patient’s neutrophils, the presence of which reflected the phenomenon of nuclear fragmentation (indicated by yellow arrows in Figure 1: E). It was worth mentioning that the activated partial thromboplastin time (APTT) and thrombin time (TT) of the patient were prolonged, while prothrombin time (PT) was normal, which suggested that there might be heparin-like substances in her blood circulation, and further suggested that there was damage to the vascular endothelium. Damaged endothelial cells release heparan sulfate, which has a heparin-like effect. It has been reported that the degree of elongation of APTT and TT induced by heparan sulfate was positively correlated with the degree of endothelial injury (*15*, *16*). There were no BGNI in her peripheral blood until 24 hours before she died. In addition, the color of BGNI deepened and the number of BGNI increased, indicating that the color and number of BGNI are directly related to the patient’s prognosis.

Wasp stings, that is, venom invasion caused by bee tail puncture to the skin, can cause a series of serious physiological reactions, including local redness, stinging, acute systemic poisoning symptoms, allergies, local toxic reactions and multi-system organ damage (*17*). Especially for severe wasp stings, the disease progresses rapidly. Once patients have multiple organ insufficiency syndrome (MODS), the mortality rate will increase significantly (*18*). According to statistics, the annual mortality rate caused by wasp stings in the United States was 0.74/1 million, while the epidemiological data in China in this regard were still insufficient (*19*). In case 2, the patient was stung by wasp swarm, and the venom quickly spread throughout the patient’s body along with the blood circulation, resulting in acute liver failure and multiple organ failure syndrome. The patient’s APTT was significantly prolonged, mainly because the venom increased vascular permeability and exposed subcutaneous tissue factors within the blood vessels, which activated the clotting pathway and consumed a large amount of clotting factors. At the same time, the activities of serum enzymes related to liver function in this patient was abnormally elevated, which may be due to the necrosis of liver cells caused by the wasp venom (*20*). In view of the above pathological mechanisms, early hemoperfusion therapy is very important for patients with wasp stings. When patients develop liver and kidney failure or rhabdomyolysis syndrome, timely plasma exchange and hemodialysis filtration are key life-saving measures (*18*, *21*, *22*). These treatments effectively remove venom from the body, thereby protecting the liver and other vital organs. In this case, it was because of the timely discovery of BGNI and the correct treatment measures, the patient was able to successfully cure.

In the two cases we reported, both of them had BGNI in their peripheral blood, but the color changes of BGNI in the two patients were different, and the prognosis of the patients was also significantly different. Case 1 infected with the novel buniavirus developed BGNI on the day of death, and in the hours leading up to their demise, the color of BGNI rapidly changed from light green to dark green. In contrast, dark green BGNI was observed on the day of admission after being stung by wasps in case 2. With the improvement of the patient’s condition, the color of BGNI becomes shallow gradually, patients eventually recovered and was discharged from the hospital.

These findings indicated a potential correlation between the color variations of BGNI and prognosis. However, it was crucial to conduct further comprehensive studies to assess this hypothesis. The laboratory should enhance peripheral blood smear examinations for critically ill patients. Upon detecting BGNI, laboratory staff must promptly inform the attending physician to initiate targeted treatments, such as hepatic support and/or acidosis correction, based on the patient’s condition.

## Data Availability

The data generated and analyzed in the presented study are available from the corresponding author on request.
